# Coupled Spreading Dynamics of Competitive Information and Epidemics on Weighted Multilayer Networks with Higher-Order Interactions

**DOI:** 10.3390/e28101138

**Published:** 2026-10-16

**Authors:** Qian Yin, Haoxian Li, Zhishuang Wang, Kaiyao Wang, Jingmin Li, Zhiyong Hong

**Affiliations:** School of Electronics and Information Engineering, Wuyi University, Jiangmen 529020, China

**Keywords:** multilayer complex networks, higher-order interactions, activity-driven networks, coupled spreading

## Abstract

The spread of epidemics in real social systems is often coupled with competitive information diffusion, higher-order group interactions, and dynamic contact behaviors. To capture these mechanisms, we develop a bilayer competitive information–epidemic coupled spreading framework. The upper layer is a weighted higher-order network incorporating both pairwise spreading and 2-simplex-driven group spreading, while the lower layer is a weighted activity-driven network describing time-varying physical contacts regulated by a policy relaxation parameter. Based on the microscopic Markov chain framework, we establish node-level evolution equations and derive a spectral condition for the local invasion threshold, which is evaluated numerically. Monte Carlo simulations show good agreement with the predicted steady-state densities. These findings indicate that enhancing the diffusion of the more protective information reduces steady-state epidemic prevalence and raises the epidemic threshold, whereas preference for the less protective information weakens the overall protective effect. Higher-order interactions exert asymmetric effects on competing information, while social tie weights and activity-driven contacts also significantly influence epidemic spreading. Moreover, the epidemic threshold is jointly affected by recovery probability, activity heterogeneity, information-layer topology, and policy regulation. These findings reveal the cross-layer coupling between higher-order information diffusion and temporal physical contacts.

## 1. Introduction

Epidemic spreading is not an isolated biological process but is closely intertwined with information acquisition, risk perception, and behavioral responses of individuals [1]. During public health emergencies in particular, disease-related information can rapidly diffuse through social interactions and subsequently influence protective decisions and contact behaviors, which in turn reshape epidemic transmission [2]. Meanwhile, real information environments usually contain multiple competing messages or viewpoints that differ in spreading capability, protective effectiveness, and individual adoption preference [3,4,5,6,7,8]. Consequently, information diffusion and epidemic spreading form a complex bidirectional coupling: epidemic prevalence can heighten public attention to disease-related content and facilitate its dissemination, while different types of information can alter protective behavior and infection risk, thereby affecting the eventual epidemic prevalence [9]. Understanding the coevolution of competitive information and epidemic spreading is therefore important for revealing spreading mechanisms in complex social systems and for designing effective public health interventions [10].

Complex networks provide an important modeling framework for investigating such coupled spreading processes [11]. In particular, multilayer complex networks can represent social information interactions and physical contact relationships in different network layers, thereby capturing cross-layer coupling between distinct spreading processes [12,13,14,15,16,17]. Moreover, real social relationships are heterogeneous in interaction strength, and weighted networks provide a natural framework for characterizing such heterogeneity [18,19,20,21,22]. However, conventional multilayer networks are generally built on pairwise interactions and may therefore be insufficient to characterize collective phenomena induced by group-level interactions [23,24]. To overcome this shortcoming, simplex-based higher-order complex networks introduce higher-order structures such as 2-simplices, providing a natural representation of group reinforcement, cooperative spreading, and collective influence [25,26,27,28,29]. Meanwhile, the physical contacts underlying epidemic transmission are inherently time-varying, and activity-driven networks can dynamically generate and update links according to individual activity levels, making them suitable for describing temporal contacts arising from commuting, working, and social activities [30,31,32]. Although multilayer complex networks, higher-order networks, and activity-driven networks capture different aspects of real spreading systems, more complex cross-layer feedback may emerge when competitive information spreading, higher-order group interactions, temporal physical contacts, and macroscopic policy intervention coexist. Therefore, integrating multilayer network structures, simplex-based non-pairwise interactions, as well as activity-driven mechanisms into an integrated framework remains necessary to achieve a more complete characterization of coupled information–disease dynamics and the conditions governing epidemic outbreaks [33,34].

For coupled spreading on simplex-based multilayer complex networks, existing studies have increasingly incorporated higher-order interactions with individual heterogeneity, risk perception, media influence, and population mobility. You et al. [35] developed a duplex multi-information–multi-disease model and showed that higher-order interactions can induce and sustain bistable epidemic dynamics, while multi-information diffusion significantly affects disease coexistence and prevalence. Li et al. [36] introduced variability in individual activity levels, information uptake, and cross-layer coupling into a higher-order multiplex framework, demonstrating that individual heterogeneity reshapes information–disease co-diffusion, with activity-dependent discontinuous phase transitions arising from higher-order interactions. Sun et al. [37] incorporated cumulative risk perception and heterogeneous thresholds into a duplex awareness–epidemic model, showing that stronger pairwise and higher-order information spreading can elevate the epidemic threshold while lowering steady-state infection prevalence. Mei et al. [38] subsequently combined non-pairwise information interactions, media influence, and population mobility, finding that media dissemination and higher-order interactions suppress epidemic spreading by enhancing public awareness, while reduced mobility and optimized inter-population connectivity further improve epidemic control. Overall, these studies demonstrate that simplex-based higher-order interactions can substantially reshape steady states, epidemic thresholds, and phase-transition behaviors in multilayer coupled spreading systems.

Alongside the growing attention to higher-order group interactions, the temporal evolution of contact relationships in real spreading systems has also attracted increasing interest, motivating the introduction of activity-driven networks into multilayer coupled spreading models [39,40,41]. Zhang et al. [42] developed a bilayer activity-driven framework for information–epidemic spreading with partial interlayer mapping and 2-simplices in the information layer, showing that higher mapping rates and stronger higher-order information transmission enhance information diffusion, raise the epidemic onset threshold while lowering infection. Huo et al. [43] developed a three-layer information–emotion–epidemic framework and found that such mutual reinforcement of negative information and anxiety enlarges epidemic prevalence and lowers the epidemic threshold, whereas suppressing anxiety transmission helps mitigate epidemic spreading. Wang et al. [44] integrated metapopulation mobility with activity-driven information dissemination and showed how information diffusion elevates the epidemic threshold and reduces disease prevalence, while media credibility, community heterogeneity, and migration preferences also play important roles. Hong et al. [45] investigated information–behavior–epidemic coevolution on adaptive multiplex temporal networks and demonstrated that adaptive rewiring can markedly raise the epidemic threshold, whereas compulsory regulation mainly reduces epidemic prevalence after outbreak. Overall, activity-driven and adaptive temporal networks further extend the framework of multilayer coupled spreading and highlight the important regulatory roles of dynamic contact structures, information feedback, and behavioral responses in epidemic dynamics.

Previous research has established important roles of information competition, weighted interactions, higher-order reinforcement, and temporal contacts in information–epidemic coevolution. Related frameworks have been extended through information coexistence, individual heterogeneity, and activity-driven interactions [35,36,42]. Building on these findings, a specific question requires further clarification: when two mutually exclusive information types provide different levels of protection against the same disease, how does weighted higher-order reinforcement change population protection through information competition, and how does this change interact with policy-modulated temporal contacts to influence epidemic spreading? In this setting, social tie weights and simplex weights affect information diffusion and replacement, while individual preference further changes the relative dominance of the two information types. The resulting information distribution determines population protection, whereas activity-driven contacts and policy regulation directly influence disease exposure. It is therefore necessary to distinguish the indirect effects of weighted higher-order interactions through the competitive information distribution from the direct effects of temporal contacts on infection opportunities, and to determine how these two pathways jointly shape the critical condition and steady-state prevalence of the epidemic.

The principal contributions of this study are threefold. First, we develop a coupled model with a weighted higher-order information layer and an activity-driven epidemic layer. Information replacement and competitive attenuation are incorporated into both pairwise and 2-simplex transmission, while individual preference determines adoption when both information types are received. Combined with their different protective effects, this formulation captures how competitive redistribution of information influences the spread of the same disease. Second, we formulate node-level microscopic Markov chain (MMC) equations and construct an effective spreading matrix incorporating the stationary distribution of competing information in the disease-free state, information-induced protection, individual activity, and policy-modulated contacts. Its spectral properties determine the local invasion condition around a selected stable disease-free information equilibrium. Information-layer weights, higher-order interactions, and competition enter this condition through the stationary information distribution, whereas individual activity and policy regulation determine the contact terms, thereby making their combined roles explicit. Third, numerical analyses and Monte Carlo (MC) simulations test the theoretical predictions and reveal specific competitive and higher-order effects. Under the examined conditions, preference for the less protective information weakens population protection, while strengthening higher-order transmission of the more protective information produces stronger epidemic suppression than strengthening that of the other information type. We further compare the effects of social tie weights and temporal contact intensity on epidemic prevalence and examine how information competition, activity heterogeneity, and policy regulation jointly shift the critical transmission probability.

The rest of the paper is organized in the following manner. Section 2 presents the bilayer coupled propagation model together with the policy intervention scheme. Section 3 formulates the governing dynamical equations using the MMC approach and derives a spectral condition for the local invasion threshold. Section 4 verifies the analytical results by means of numerical simulations and examines the influence of key parameters on spreading behavior. Section 5 concludes the paper.

## 2. Coupled Propagation Model

### 2.1. Bilayer Network Structure

**Figure 1:**
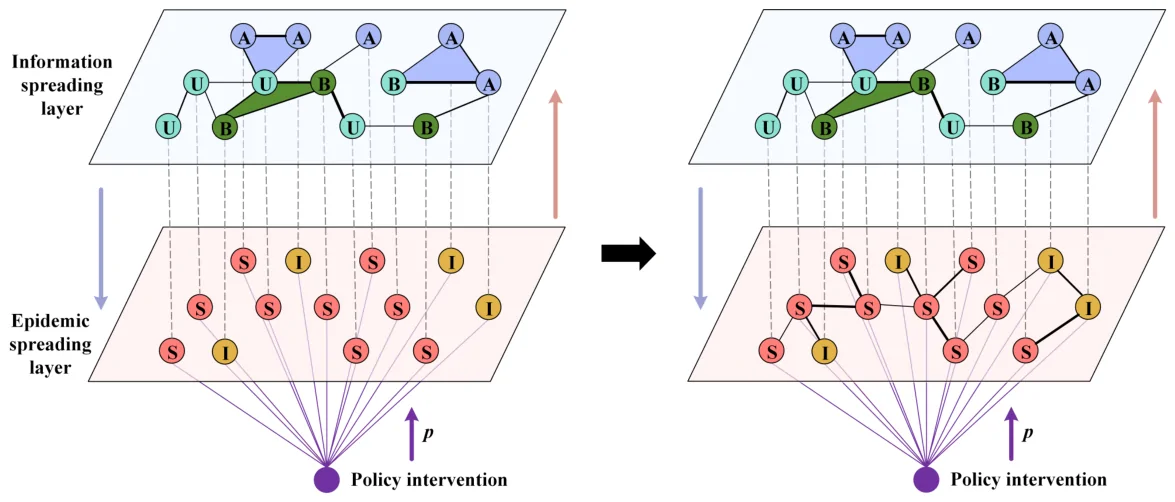
Schematic representation of the developed bilayer coupled spreading framework under policy intervention. The upper layer represents competitive information spreading on a weighted higher-order network, where nodes can be in states U, A, or B, and the shaded triangles denote 2-simplices. Epidemic spreading in the lower layer takes place over a weighted activity-driven network, where each node occupies either the S or I state, while contact links evolve over time. Dashed lines indicate the one-to-one mapping of counterpart nodes across the two layers. The policy relaxation parameter *p* regulates contact weights in the epidemic layer, while the two layers mutually influence the coupled spreading dynamics.

To characterize the coupled spreading of two competing information types together with an epidemic under dynamically changing contacts, we construct a bilayer network consisting of one layer for information spreading and another for epidemic transmission, as illustrated in Figure 1. The two layers contain the same number of nodes and are coupled through a one-to-one correspondence, such that counterpart nodes represent the same individual. The upper layer describes information interactions among individuals, whereas the lower layer represents epidemic transmission through physical contacts.

The upper information layer takes the form of a weighted higher-order structure. In addition to conventional pairwise interactions, 2-simplices composed of three nodes are introduced to represent collective effects generated by group interactions. To account for the heterogeneity of social relationships, different weights are assigned to network links. Specifically, the weight assigned to the link connecting nodes *i* and *j*, represented by $a_{ij}$, satisfies

(1) $$a_{ij}∼N\left(\mu_{a},\sigma_{a}^{2}\right),\;0\leq a_{ij}\leq 1,$$

where $\mu_{a}$ and $\sigma_{a}$ are the location and scale parameters of the normal distribution before truncation, respectively. A larger $a_{ij}$ indicates a stronger social relationship and hence a greater intensity of information interaction.

The lower epidemic layer is modeled as a weighted activity-driven network. Unlike networks with fixed topologies, the activity-driven mechanism allows links to be dynamically created and removed over time, representing transient physical contacts arising from commuting, working, and social activities. At each time step, nodes establish temporary links according to their intrinsic activity levels, and the network topology is renewed in the subsequent step. Let $b_{i}$ denote the probability that node *i* is activated and initiates contacts during each model update step. We set $b_{i}=\eta_{R}y_{i}$, where $y_{i}\in \left[y_{\min},1\right]$ represents the individual’s underlying activity propensity and $\eta_{R}$ controls the overall activation level, subject to $0\leq \eta_{R}y_{i}\leq 1$. An inactive node may still be selected as a contact partner by another activated node. The numerical activity probabilities depend on the chosen time-step duration and would require contact observations for their determination in practical applications. The expected link intensity between nodes *i* and *j* can be expressed as

(2) $$b_{ij}=\frac{m_{R}}{N-1}\left(b_{i}+b_{j}\right),$$

where $m_{R}$ denotes the expected count of links established by an activated node within one update step, and *N* is the network size. The expected-link matrix $\left[b_{ij}\right]$, composed of all $b_{ij}$, characterizes the expected contact intensity between each pair of nodes under the activity-driven mechanism, with $b_{ii}=0$. It therefore provides an effective representation of the time-averaged contact structure of the epidemic layer. On this basis, the effective contact weights in the epidemic layer are further regulated by macroscopic policy intervention, thereby influencing epidemic transmission.

Thus, the bilayer framework simultaneously incorporates higher-order information interactions, heterogeneous social relationships, temporal physical contacts, and policy intervention into the coupled spreading dynamics. Within this framework, the topology, 2-simplex configurations, and edge weights of the information layer remain fixed during the dynamical evolution, whereas temporary contact links in the epidemic layer are renewed at each time step according to the activity-driven mechanism. The activation probabilities and contact-partner selection rules are prescribed in the present model, without adaptive feedback through which individuals adjust their activities or contact relationships in response to their information states or infection risks.

### 2.2. Information-Spreading Layer

**Figure 2:**
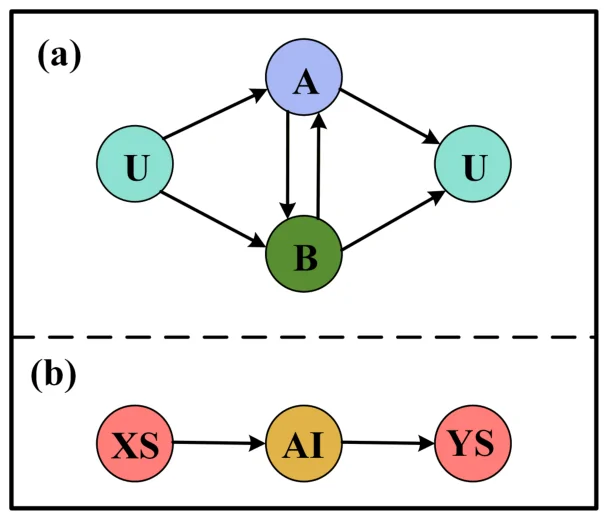
Schematic illustration of the state-transition processes for the coupled propagation framework. (**a**) Transition pathways among the unaware state U and the two competing information states A and B in the information-spreading layer. (**b**) Epidemic-state transition processes modulated by information in the epidemic-spreading layer, where X∈{U,A,B} and Y∈{U,A}.

In the information-spreading layer, a UABU competitive spreading mechanism is employed to characterize the evolution of two competing information types on a weighted higher-order network. Each individual can occupy one of three possible states: unaware (U), informed by information A (A), or informed by information B (B). Information propagation proceeds through pairwise contacts as well as higher-order group interactions. Figure 2a summarizes the information-state transitions, while Figure 2b illustrates the coupling between information states and the infection–recovery process. Here, XS denotes a susceptible individual with information state X∈{U,A,B}. Upon infection, the individual adopts information A and enters AI. Following recovery, the individual enters AS or US depending on whether information A is retained, so that Y∈{U,A} in YS. The coupling mechanism illustrated in Figure 2b is explained in greater detail in Section 2.3.

For pairwise spreading, the state transitions are illustrated in Figure 2a. Nodes occupying states A and B convey the corresponding information to unaware neighbors with probabilities $\lambda_{A}$ and $\lambda_{B}$, respectively. To characterize the competitive exclusion between the two types of information, a competition attenuation factor η∈[0,1] is introduced. When the target individual already holds the competing information, the effective transmission probabilities are reduced to $\eta \lambda_{A}$ and $\eta \lambda_{B}$, respectively. In addition, individuals in states A and B lose the corresponding information and revert to state U with probabilities $\delta_{A}$ and $\delta_{B}$.

**Figure 3:**
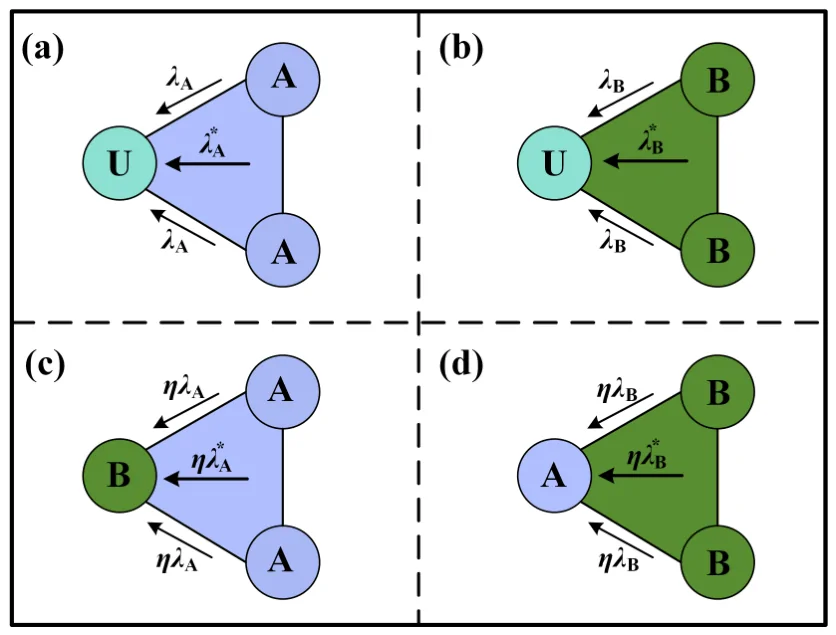
Schematic illustration of higher-order information spreading through 2-simplices. (**a**) An unaware node U receives information A from two A-state neighbors through pairwise transmission with probability $\lambda_{A}$ and higher-order transmission with probability $\lambda_{A}^{*}$. (**b**) The corresponding spreading process for information B with probabilities $\lambda_{B}$ and $\lambda_{B}^{*}$. (**c**) A B-state node receives competing information A, where the pairwise and higher-order transmission probabilities are reduced to $\eta \lambda_{A}$ and $\eta \lambda_{A}^{*}$, respectively. (**d**) The corresponding competitive spreading of information B to an A-state node, with probabilities $\eta \lambda_{B}$ and $\eta \lambda_{B}^{*}$.

Beyond pairwise spreading, higher-order transmission driven by 2-simplices is further considered, as illustrated in Figure 3. When an unaware individual belongs to a 2-simplex with two A-state individuals, information A is transmitted with probability $\lambda_{A}^{*}$. Similarly, the higher-order transmission probability of information B is $\lambda_{B}^{*}$. If the target individual already holds the competing information, the corresponding higher-order transmission probabilities are reduced to $\eta \lambda_{A}^{*}$ and $\eta \lambda_{B}^{*}$, respectively.

To account for heterogeneous social relationships within a 2-simplex, the edge set of the simplex composed of nodes *i*, *j*, and *k* can be written as $E_{ijk}=\left\{\left(i,j\right),\left(i,k\right),\left(j,k\right)\right\}$. The comprehensive influence weight of the 2-simplex is then given by

(3) $$\omega_{ijk}=\frac{1}{|E_{ijk}|}\sum_{\left(u,v\right)\in E_{ijk}}a_{uv},$$

where $a_{uv}$ is the weight associated with the edge connecting nodes *u* and *v*. This quantity captures the overall strength of pairwise social relationships within the 2-simplex and characterizes heterogeneous group influence on higher-order information spreading.

### 2.3. Epidemic-Spreading Layer

In the epidemic-spreading layer, disease transmission through dynamically changing physical contacts is modeled using the susceptible–infected–susceptible (SIS) framework. Each individual occupies either the susceptible (S) state or infected (I) state, and the associated transitions are illustrated in Figure 2b. Effective interaction with an infected neighbor may cause a susceptible individual to become infected, whereas recovery returns an infected individual to the susceptible state with probability μ.

Owing to the one-to-one correspondence between the two layers, an individual’s information state further modulates its infection risk. For an individual in the unaware state U, the baseline infection probability is $\beta^{U}=\beta$. When an individual is in state A or B, the corresponding information promotes different levels of protective behavior, reducing the infection probabilities to $\beta^{A}=\gamma_{A}\beta$, $\beta^{B}=\gamma_{B}\beta$, where $\gamma_{A}$ and $\gamma_{B}$ denote the disease attenuation factors associated with information A and B, respectively, and satisfy $0<\gamma_{A}<\gamma_{B}\leq 1$. Therefore, information A provides stronger protection than information B, establishing a cross-layer modulation of epidemic transmission by information spreading. The two information states can be interpreted as the adoption of competing disease-related recommendations supporting different precautionary practices, such as more comprehensive or less comprehensive protective measures. The model examines how the adoption and competitive replacement of these recommendations change population protection, with their protective effects represented by the disease attenuation factors.

Combining the states of the information and epidemic layers yields several possible joint states. The model assumes that individuals become aware of their own infection immediately upon infection, adopt the more protective information A within the same update step, and retain it while infected. This treatment builds on the infection-induced awareness mechanism in ref. [12] and further assigns information A as the infection-induced response state. The assumption approximates spreading settings in which infection can be readily perceived by infected individuals and their social neighbors, as may occur in cases of influenza with noticeable manifestations. For infected individuals, personal infection provides a direct risk signal that prompts reassessment of disease risk and prioritization of the more protective information. For their neighbors, infection within their social surroundings signals potential disease exposure and may increase their willingness to seek protective information and strengthen precautions. In the model, infected individuals therefore act as sources of information A, which their neighbors receive through the existing information-spreading mechanisms, producing feedback from infection to protective information dissemination. Delays in recognizing infection and adopting information are neglected to capture this rapid response. Therefore, the UI and BI states are neglected in the dynamical evolution. Consequently, four effective joint states are retained: unaware-susceptible (US), information-A-susceptible (AS), information-B-susceptible (BS), and information-A-infected (AI).

### 2.4. Policy Regulation

To characterize the regulation of individual contact behavior and epidemic transmission by macroscopic public health policies, a policy relaxation parameter *p* (p≥0) is introduced into the epidemic-spreading layer. This parameter represents the degree to which contact restrictions are relaxed, with larger *p* corresponding to stronger effective contacts during the restoration of socioeconomic activities. Rather than directly changing the spreading state of individuals, policy regulation regulates the contact weights of the activity-driven network and thereby modifies effective physical contacts.

Specifically, a policy regulation factor $v_{ij}\in \left[0,1\right]$ is introduced and assumed to follow

(4) $$v_{ij}∼U\left(0,v\left(p\right)\right),$$

where v(p) specifies the upper bound of the policy weight $v_{ij}$. For a given *p*, $v_{ij}$ is sampled from the above uniform distribution, with mean v(p)/2. The policy weight modulates the contribution of contacts between a node pair to infection exposure, and its product with $b_{ij}$, namely $v_{ij}b_{ij}$, gives the corresponding effective contact intensity. In this model, policy regulation acts through contact weights and does not directly change the activation probabilities $b_{i}$. The upper-bound function v(p) is defined as

(5) $$v\left(p\right)=\frac{2}{1+\exp(-p)}-1.$$

This function is used as a simplified nonlinear mapping between policy relaxation and contact weights, motivated by differences in individual responsiveness to policy relaxation. Because of different activity requirements and behavioral preferences, individuals may adjust their activities to different extents under the same relaxation level. The smooth response approximates the aggregate effect of these differences on contact weights. Saturation reflects a simplifying assumption that further policy relaxation yields progressively smaller increases in effective contacts as routine activities are restored. In the model, this is represented by the contact-weight upper bound v(p) gradually approaching its normalized limit of 1 as *p* increases. In comparison, a linear mapping assumes a constant aggregate marginal response, whereas a step mapping represents an abrupt aggregate change; the selected form captures diminishing marginal increments while keeping contact weights bounded. Accordingly, the effective contact intensity for a node pair *i* and *j* depends jointly on the expected link intensity $b_{ij}$ generated by the activity-driven mechanism and the policy regulation factor $v_{ij}$, thereby mapping macroscopic policy intervention onto microscopic contact processes.

Under the combined effects of policy intervention and information-induced protection, a susceptible node *i* has different actual infection probabilities depending on whether its information state is U, A, or B. Upon contact with an infected node *j*, these probabilities are given by ${\tilde{\beta }}^{U}=1-{\left(1-\beta \right)}^{v_{ij}b_{ij}}$, ${\tilde{\beta }}^{A}=1-{\left(1-\beta^{A}\right)}^{v_{ij}b_{ij}}$, ${\tilde{\beta }}^{B}=1-{\left(1-\beta^{B}\right)}^{v_{ij}b_{ij}}$. These expressions integrate information-induced protection, temporal contact intensity, and policy regulation into a unified infection process.

## 3. Theoretical Analysis

### 3.1. Dynamical Equations of the Coupled Propagation Model

The temporal evolution of the coupled competitive information–epidemic process under policy intervention is described using the MMC approach. For each node *i*, we define $p_{i}^{\mathrm{US}}\left(t\right)$, $p_{i}^{\mathrm{AS}}\left(t\right)$, $p_{i}^{\mathrm{BS}}\left(t\right)$, and $p_{i}^{\mathrm{AI}}\left(t\right)$ as the probabilities of occupying the four effective joint states US, AS, BS, and AI, respectively, at time *t*. Their sum is constrained by

(6) $$p_{i}^{\mathrm{US}}\left(t\right)+p_{i}^{\mathrm{AS}}\left(t\right)+p_{i}^{\mathrm{BS}}\left(t\right)+p_{i}^{\mathrm{AI}}\left(t\right)=1.$$

Accordingly, the marginal probabilities associated with information states A and B for node *i* can be expressed as

(7) $$p_{i}^{A}\left(t\right)=p_{i}^{\mathrm{AS}}\left(t\right)+p_{i}^{\mathrm{AI}}\left(t\right),\;p_{i}^{B}\left(t\right)=p_{i}^{\mathrm{BS}}\left(t\right).$$

We first consider information spreading through pairwise interactions. For a node *i* in state U, let $r_{i}^{A}\left(t\right)$ and $r_{i}^{B}\left(t\right)$ denote the probabilities that it receives no information A or B, respectively, via pairwise contacts with its neighbors during time step *t*. They are given by

(8) $$\left\{\begin{array}{cc} r_{i}^{A}\left(t\right) & =\prod_{j}\left[1-a_{ji}p_{j}^{A}\left(t\right)\lambda_{A}\right],\\ r_{i}^{B}\left(t\right) & =\prod_{j}\left[1-a_{ji}p_{j}^{B}\left(t\right)\lambda_{B}\right]. \end{array}\right.$$

where $a_{ji}$ denotes the weight assigned to the connection between nodes *j* and *i* on the information layer. It follows that $1-r_{i}^{A}\left(t\right)$ and $1-r_{i}^{B}\left(t\right)$ give the probabilities of information A and B, respectively, reaching node *i* through at least one neighbor.

When a node already holds the competing information, its adoption of the alternative information is suppressed by the competition attenuation factor η. Let $s_{i}^{A}\left(t\right)$ denote the probability that none of the A-state neighbors successfully influences a node currently in state B, while $s_{i}^{B}\left(t\right)$ is defined analogously for a node in state A when exposed to information B. These probabilities are expressed as

(9) $$\left\{\begin{array}{cc} s_{i}^{A}\left(t\right) & =\prod_{j}\left[1-a_{ji}p_{j}^{A}\left(t\right)\eta \lambda_{A}\right],\\ s_{i}^{B}\left(t\right) & =\prod_{j}\left[1-a_{ji}p_{j}^{B}\left(t\right)\eta \lambda_{B}\right]. \end{array}\right.$$

Thus, the attenuation factor η explicitly captures the suppression of competing information by an individual’s existing information state.

In addition to pairwise interactions, higher-order transmission through 2-simplices is incorporated. Let $c_{ijk}$ be a simplex indicator, taking the value 1 when nodes *i*, *j*, and *k* constitute a 2-simplex and 0 otherwise. For a U-state node *i*, $\phi_{i}^{A|U}\left(t\right)$ and $\phi_{i}^{B|U}\left(t\right)$ represent the probabilities that it escapes all higher-order exposure to information A and B, respectively.

(10) $$\left\{\begin{array}{cc} \phi_{i}^{A|U}\left(t\right) & =\prod_{j<k}\left[1-c_{ijk}\omega_{ijk}p_{j}^{A}\left(t\right)p_{k}^{A}\left(t\right)\lambda_{A}^{*}\right],\\ \phi_{i}^{B|U}\left(t\right) & =\prod_{j<k}\left[1-c_{ijk}\omega_{ijk}p_{j}^{B}\left(t\right)p_{k}^{B}\left(t\right)\lambda_{B}^{*}\right]. \end{array}\right.$$

where $\omega_{ijk}$ quantifies the overall influence associated with the 2-simplex involving nodes *i*, *j*, and *k*. These expressions indicate that a higher-order transmission event is induced when the other two nodes within the same 2-simplex simultaneously carry the same type of information.

Similarly, when the target node already carries the competing information, higher-order transmission is attenuated by η. The corresponding escape probabilities take the following form:

(11) $$\left\{\begin{array}{cc} \phi_{i}^{A|B}\left(t\right) & =\prod_{j<k}\left[1-c_{ijk}\omega_{ijk}p_{j}^{A}\left(t\right)p_{k}^{A}\left(t\right)\eta \lambda_{A}^{*}\right],\\ \phi_{i}^{B|A}\left(t\right) & =\prod_{j<k}\left[1-c_{ijk}\omega_{ijk}p_{j}^{B}\left(t\right)p_{k}^{B}\left(t\right)\eta \lambda_{B}^{*}\right]. \end{array}\right.$$

The combined contributions of pairwise and higher-order spreading determine the transition probabilities of node *i* among different information states. For a node in state U, let $\theta_{i}^{U|U}\left(t\right)$ denote the probability that it receives neither information A nor information B during time step *t* and thus remains in state U. Similarly, $\theta_{i}^{A|U}\left(t\right)$ and $\theta_{i}^{B|U}\left(t\right)$ represent the probabilities of a U-state node switching to states A and B, respectively. These probabilities are given by

(12) $$\left\{\begin{array}{cc} \theta_{i}^{U|U}\left(t\right) & =r_{i}^{A}\left(t\right)\phi_{i}^{A|U}\left(t\right)r_{i}^{B}\left(t\right)\phi_{i}^{B|U}\left(t\right),\\ \theta_{i}^{A|U}\left(t\right) & =1-r_{i}^{A}\left(t\right)\phi_{i}^{A|U}\left(t\right)-\alpha \left[1-r_{i}^{A}\left(t\right)\phi_{i}^{A|U}\left(t\right)\right]\left[1-r_{i}^{B}\left(t\right)\phi_{i}^{B|U}\left(t\right)\right],\\ \theta_{i}^{B|U}\left(t\right) & =1-r_{i}^{B}\left(t\right)\phi_{i}^{B|U}\left(t\right)-\left(1-\alpha \right)\left[1-r_{i}^{A}\left(t\right)\phi_{i}^{A|U}\left(t\right)\right]\left[1-r_{i}^{B}\left(t\right)\phi_{i}^{B|U}\left(t\right)\right]. \end{array}\right.$$

Here, α (0≤α≤1) characterizes the preference between the two competing types of information when a node is simultaneously exposed to both. In such a case, the node adopts information B with probability α and information A with probability 1−α. Thus, a larger α corresponds to a stronger relative preference for information B.

For nodes that already hold information A or B, state switching is jointly determined by the combined influence of pairwise and higher-order spreading of the competing information. Let $\theta_{i}^{A|B}\left(t\right)$ and $\theta_{i}^{B|A}\left(t\right)$ represent the transition probabilities from B to A and from A to B, respectively. These quantities can be written as

(13) $$\left\{\begin{array}{cc} \theta_{i}^{A|B}\left(t\right) & =1-s_{i}^{A}\left(t\right)\phi_{i}^{A|B}\left(t\right),\\ \theta_{i}^{B|A}\left(t\right) & =1-s_{i}^{B}\left(t\right)\phi_{i}^{B|A}\left(t\right). \end{array}\right.$$

These transition probabilities jointly account for competitive attenuation, heterogeneous pairwise contacts, together with collective higher-order effects, thereby describing microscopic information-state switching at the information-spreading layer.

We next consider the epidemic-spreading layer. Since different information states induce different levels of protective behavior, susceptible nodes in states U, A, and B experience different infection risks. Let $q_{i}^{U}\left(t\right)$, $q_{i}^{A}\left(t\right)$, and $q_{i}^{B}\left(t\right)$ represent the probabilities that no infected neighbor successfully transmits the disease to node *i* during time step *t*, conditional on its respective information state. Their expressions are

(14) $$\left\{\begin{array}{cc} q_{i}^{U}\left(t\right) & =\prod_{j}\left[1-{\tilde{\beta }}^{U}p_{j}^{I}\left(t\right)\right],\\ q_{i}^{A}\left(t\right) & =\prod_{j}\left[1-{\tilde{\beta }}^{A}p_{j}^{I}\left(t\right)\right],\\ q_{i}^{B}\left(t\right) & =\prod_{j}\left[1-{\tilde{\beta }}^{B}p_{j}^{I}\left(t\right)\right]. \end{array}\right.$$

where $p_{j}^{I}\left(t\right)=p_{j}^{\mathrm{AI}}\left(t\right)$ represents the infection probability of node *j* at time *t*, ${\tilde{\beta }}^{U}$, ${\tilde{\beta }}^{A}$, and ${\tilde{\beta }}^{B}$ represent the state-dependent effective infection probabilities obtained after accounting for dynamic contacts and policy intervention. Consequently, $1-q_{i}^{U}\left(t\right)$, $1-q_{i}^{A}\left(t\right)$, and $1-q_{i}^{B}\left(t\right)$ represent the probabilities of successful disease transmission to node *i* from one or more infected neighbors under the three information states, respectively.

**Figure 4:**
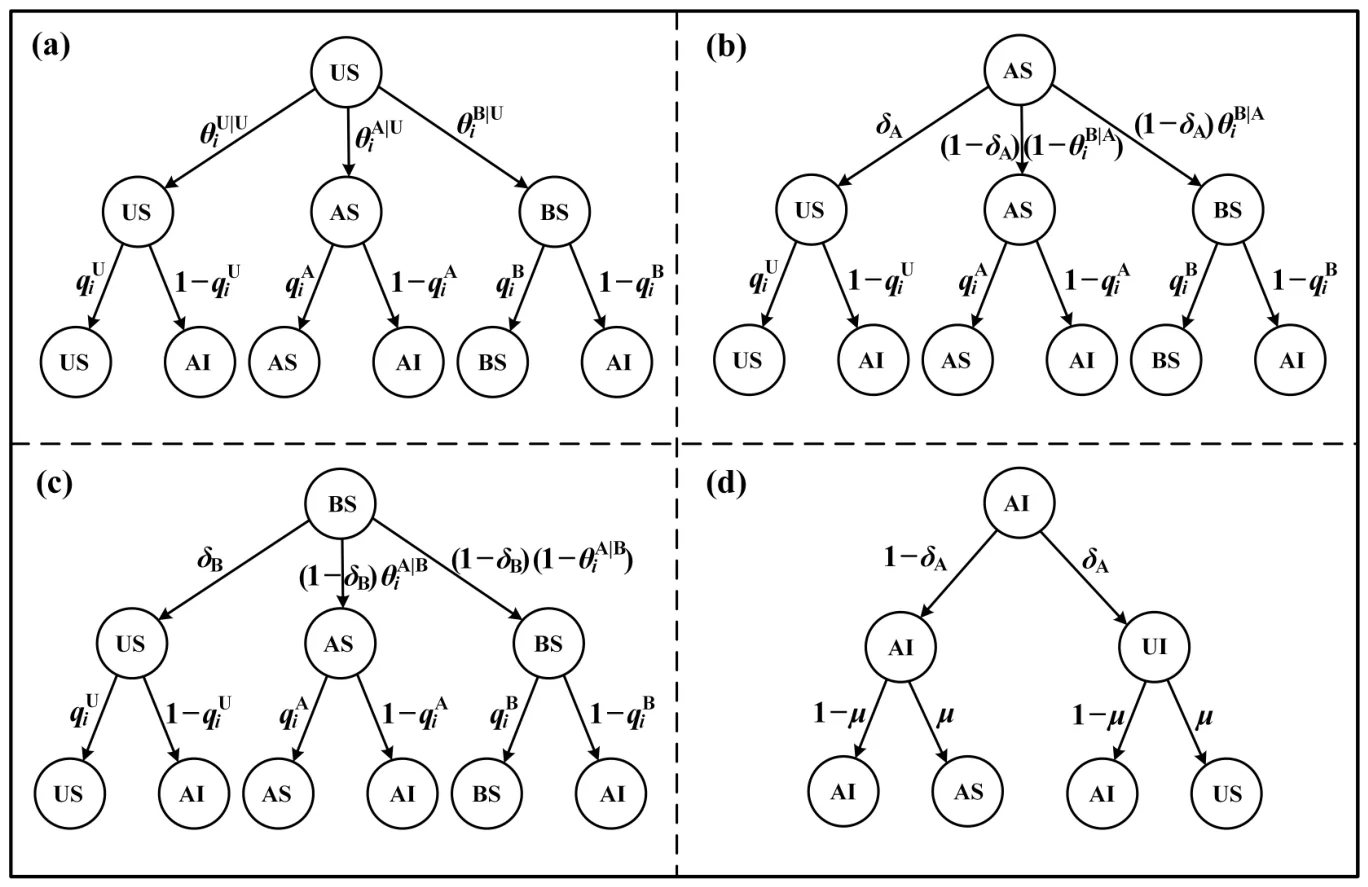
State-transition trees for the four effective joint states in the coupled propagation model. (**a**) Transitions starting from the unaware-susceptible state US. (**b**) Transitions starting from the information-A-susceptible state AS. (**c**) Transitions starting from the information-B-susceptible state BS. (**d**) Transitions starting from the information-A-infected state AI. Each branch represents a possible state transition within one time step, and the corresponding labels denote the transition probabilities determined by information spreading, information forgetting, epidemic infection, and recovery.

Based on the information- and epidemic-spreading probabilities defined above, the microscopic transition processes among the four effective joint states can be established. As depicted in Figure 4, four transition trees originate from US, AS, BS, and AI, respectively. The branches represent possible information-state and epidemic-state transitions within one time step, while the associated probabilities quantify the corresponding transition events. Under the rapid infection-induced information-response assumption specified in Section 2.3, the intermediate states UI and BI that may temporarily arise during the transition process are immediately mapped to state AI and are not retained as independent dynamical states. The consequences of relaxing this assumption can be understood by comparing the infection branches. At the disease-free steady state, these branches are inactive. When infection persists, allowing a susceptible individual retaining information B to enter BI rather than AI would redistribute the infection probability without directly changing its total value in that update. However, the BI individual could continue to disseminate information B, reducing infection-induced sources of information A. Conditional on retaining the corresponding information during recovery, the original pathway returns the individual to AS, whereas the extended pathway can return it to BS. Since $\gamma_{A}<\gamma_{B}$, the latter individual faces a higher reinfection risk under otherwise identical contact conditions. These pathways could weaken protective information feedback and increase steady-state infection prevalence, although the overall change depends on the coupled information dynamics.

Using the transition processes depicted in Figure 4 together with the previously defined information-transition and infection-escape probabilities, we obtain the MMC evolution equations governing the four effective joint states as follows:

(15) $$\left\{\begin{array}{cc} p_{i}^{\mathrm{US}}\left(t+1\right)= & p_{i}^{\mathrm{US}}\left(t\right)\theta_{i}^{U|U}\left(t\right)q_{i}^{U}\left(t\right)+p_{i}^{\mathrm{AS}}\left(t\right)\delta_{A}q_{i}^{U}\left(t\right)\\ \; & +p_{i}^{\mathrm{BS}}\left(t\right)\delta_{B}q_{i}^{U}\left(t\right)+p_{i}^{\mathrm{AI}}\left(t\right)\delta_{A}\mu ,\\ p_{i}^{\mathrm{AS}}\left(t+1\right)= & p_{i}^{\mathrm{US}}\left(t\right)\theta_{i}^{A|U}\left(t\right)q_{i}^{A}\left(t\right)\\ \; & +p_{i}^{\mathrm{AS}}\left(t\right)\left(1-\delta_{A}\right)\left[1-\theta_{i}^{B|A}\left(t\right)\right]q_{i}^{A}\left(t\right)\\ \; & +p_{i}^{\mathrm{BS}}\left(t\right)\left(1-\delta_{B}\right)\theta_{i}^{A|B}\left(t\right)q_{i}^{A}\left(t\right)\\ \; & +p_{i}^{\mathrm{AI}}\left(t\right)\left(1-\delta_{A}\right)\mu ,\\ p_{i}^{\mathrm{BS}}\left(t+1\right)= & p_{i}^{\mathrm{US}}\left(t\right)\theta_{i}^{B|U}\left(t\right)q_{i}^{B}\left(t\right)\\ \; & +p_{i}^{\mathrm{AS}}\left(t\right)\left(1-\delta_{A}\right)\theta_{i}^{B|A}\left(t\right)q_{i}^{B}\left(t\right)\\ \; & +p_{i}^{\mathrm{BS}}\left(t\right)\left(1-\delta_{B}\right)\left[1-\theta_{i}^{A|B}\left(t\right)\right]q_{i}^{B}\left(t\right),\\ p_{i}^{\mathrm{AI}}\left(t+1\right)= & p_{i}^{\mathrm{US}}\left(t\right)[\theta_{i}^{U|U}\left(t\right)\left(1-q_{i}^{U}\left(t\right)\right)+\theta_{i}^{A|U}\left(t\right)\left(1-q_{i}^{A}\left(t\right)\right)\\ \; & \;\;+\theta_{i}^{B|U}\left(t\right)\left(1-q_{i}^{B}\left(t\right)\right)]\\ \; & +p_{i}^{\mathrm{AS}}\left(t\right)[\delta_{A}\left(1-q_{i}^{U}\left(t\right)\right)+\left(1-\delta_{A}\right)\left[1-\theta_{i}^{B|A}\left(t\right)\right]\left(1-q_{i}^{A}\left(t\right)\right)\\ \; & \;\;+\left(1-\delta_{A}\right)\theta_{i}^{B|A}\left(t\right)\left(1-q_{i}^{B}\left(t\right)\right)]\\ \; & +p_{i}^{\mathrm{BS}}\left(t\right)[\delta_{B}\left(1-q_{i}^{U}\left(t\right)\right)+\left(1-\delta_{B}\right)\theta_{i}^{A|B}\left(t\right)\left(1-q_{i}^{A}\left(t\right)\right)\\ \; & \;\;+\left(1-\delta_{B}\right)\left[1-\theta_{i}^{A|B}\left(t\right)\right]\left(1-q_{i}^{B}\left(t\right)\right)]\\ \; & +p_{i}^{\mathrm{AI}}\left(t\right)\left(1-\mu \right). \end{array}\right.$$

For a given network and a fixed parameter set, steady-state densities are computed by synchronously iterating the node-state probabilities in Equation (15). Let *E* and $S_{2}$ denote the numbers of information-layer edges and 2-simplices, respectively, and let $L_{\mathrm{dyn}}$ be the number of evolution steps required for the steady-state calculation. Using adjacency and simplex lists, each information update requires $O(N+E+S_{2})$ operations. The present implementation uses a dense expected contact structure, so each epidemic update visits node pairs and requires $O(N^{2})$ operations. The time complexity for a single parameter set is therefore $O\;\left[L_{\mathrm{dyn}}\left(N^{2}+E+S_{2}\right)\right]$. When the mean edge degree and mean simplex participation degree are held fixed, $E,S_{2}=O\left(N\right)$, and the leading time complexity is $O(L_{\mathrm{dyn}}N^{2})$. The value of $L_{\mathrm{dyn}}$ depends on the model parameters and the convergence criterion.

### 3.2. Epidemic Threshold Analysis

The local invasion threshold marks the loss of linear stability of a selected stable disease-free information equilibrium to infection perturbations. Based on the MMC equations derived above, once the system converges to stationarity, the probabilities associated with the joint configurations no longer vary with time and obey

(16) $$\left\{\begin{array}{cc} p_{i}^{\mathrm{US}}\left(t+1\right) & =p_{i}^{\mathrm{US}}\left(t\right)=p_{i}^{\mathrm{US}},\\ p_{i}^{\mathrm{AS}}\left(t+1\right) & =p_{i}^{\mathrm{AS}}\left(t\right)=p_{i}^{\mathrm{AS}},\\ p_{i}^{\mathrm{BS}}\left(t+1\right) & =p_{i}^{\mathrm{BS}}\left(t\right)=p_{i}^{\mathrm{BS}},\\ p_{i}^{\mathrm{AI}}\left(t+1\right) & =p_{i}^{\mathrm{AI}}\left(t\right)=p_{i}^{\mathrm{AI}}. \end{array}\right.$$

Accordingly, the information-transition and infection-escape probabilities defined above also converge to their stationary values.

For the local invasion analysis around a stable disease-free information equilibrium, we consider infinitesimal infection probabilities and write

(17) $$p_{i}^{I}=p_{i}^{\mathrm{AI}}=\epsilon_{i},\;\epsilon_{i}\ll 1.$$

In the threshold analysis, linearization with respect to infection probabilities should be distinguished from an additional approximation with respect to the transmission probability. Let $w_{ji}=v_{ji}b_{ji}$ and write the pairwise infection probability as

$$T_{ji}^{X}\left(\beta \right)=1-{\left(1-\gamma_{X}\beta \right)}^{w_{ji}},\;X\in \left\{U,A,B\right\},$$

where $\gamma_{U}=1$, $\gamma_{A}=\gamma_{A}$, and $\gamma_{B}=\gamma_{B}$. Expanding in β gives

$$T_{ji}^{X}\left(\beta \right)=\gamma_{X}\beta w_{ji}+\frac{w_{ji}\left(1-w_{ji}\right)}{2}{\left(\gamma_{X}\beta \right)}^{2}+O\left(\beta^{3}\right).$$

Thus, replacing this infection probability by $\gamma_{X}\beta w_{ji}$ additionally involves a small-transmission-probability approximation. Since vanishing infection probabilities near the disease-free equilibrium do not require the critical transmission probability to vanish, we retain the full expression for $T_{ji}^{X}\left(\beta \right)$ and expand only to first order in the infection perturbations $\epsilon_{j}$. The infection-escape probabilities become

(18) $$q_{i}^{X}=\prod_{j}\left[1-T_{ji}^{X}\left(\beta \right)\epsilon_{j}\right]=1-\tau_{i}^{X}+{O(∥\epsilon ∥}^{2}),\;X\in \left\{U,A,B\right\}.$$

Here,

(19) $$\tau_{i}^{X}=\sum_{j}T_{ji}^{X}\left(\beta \right)\epsilon_{j},\;X\in \left\{U,A,B\right\}.$$

The quantity $\tau_{i}^{X}$ represents the first-order infection risk of node *i* in information state *X*, incorporating information-induced protection, temporal contact intensity, and policy regulation.

Substituting these expressions into the stationary MMC equations and retaining terms up to first order in the infection perturbations yields

(20) $$\left\{\begin{array}{cc} p_{i}^{\mathrm{US}}= & p_{i}^{\mathrm{US}}\theta_{i}^{U|U}\left(1-\tau_{i}^{U}\right)+p_{i}^{\mathrm{AS}}\delta_{A}\left(1-\tau_{i}^{U}\right)+p_{i}^{\mathrm{BS}}\delta_{B}\left(1-\tau_{i}^{U}\right)+\epsilon_{i}\delta_{A}\mu ,\\ p_{i}^{\mathrm{AS}}= & p_{i}^{\mathrm{US}}\theta_{i}^{A|U}\left(1-\tau_{i}^{A}\right)+p_{i}^{\mathrm{AS}}\left(1-\delta_{A}\right)\left(1-\theta_{i}^{B|A}\right)\left(1-\tau_{i}^{A}\right)\\ \; & +p_{i}^{\mathrm{BS}}\left(1-\delta_{B}\right)\theta_{i}^{A|B}\left(1-\tau_{i}^{A}\right)+\epsilon_{i}\left(1-\delta_{A}\right)\mu ,\\ p_{i}^{\mathrm{BS}}= & p_{i}^{\mathrm{US}}\theta_{i}^{B|U}\left(1-\tau_{i}^{B}\right)+p_{i}^{\mathrm{AS}}\left(1-\delta_{A}\right)\theta_{i}^{B|A}\left(1-\tau_{i}^{B}\right)\\ \; & +p_{i}^{\mathrm{BS}}\left(1-\delta_{B}\right)\left(1-\theta_{i}^{A|B}\right)\left(1-\tau_{i}^{B}\right),\\ \epsilon_{i}= & p_{i}^{\mathrm{US}}\left[\theta_{i}^{U|U}\tau_{i}^{U}+\theta_{i}^{A|U}\tau_{i}^{A}+\theta_{i}^{B|U}\tau_{i}^{B}\right]\\ \; & +p_{i}^{\mathrm{AS}}\left[\delta_{A}\tau_{i}^{U}+\left(1-\delta_{A}\right)\left(1-\theta_{i}^{B|A}\right)\tau_{i}^{A}+\left(1-\delta_{A}\right)\theta_{i}^{B|A}\tau_{i}^{B}\right]\\ \; & +p_{i}^{\mathrm{BS}}\left[\delta_{B}\tau_{i}^{U}+\left(1-\delta_{B}\right)\theta_{i}^{A|B}\tau_{i}^{A}+\left(1-\delta_{B}\right)\left(1-\theta_{i}^{A|B}\right)\tau_{i}^{B}\right]+\epsilon_{i}\left(1-\mu \right). \end{array}\right.$$

To determine the information background for the linear invasion analysis, we set all infection probabilities to zero. The stationary relations among US, AS, and BS then reduce to

(21) $$\left\{\begin{array}{cc} p_{i}^{\mathrm{US}}= & p_{i}^{\mathrm{US}}\theta_{i}^{U|U}+p_{i}^{\mathrm{AS}}\delta_{A}+p_{i}^{\mathrm{BS}}\delta_{B},\\ p_{i}^{\mathrm{AS}}= & p_{i}^{\mathrm{US}}\theta_{i}^{A|U}+p_{i}^{\mathrm{AS}}\left(1-\delta_{A}\right)\left(1-\theta_{i}^{B|A}\right)+p_{i}^{\mathrm{BS}}\left(1-\delta_{B}\right)\theta_{i}^{A|B},\\ p_{i}^{\mathrm{BS}}= & p_{i}^{\mathrm{US}}\theta_{i}^{B|U}+p_{i}^{\mathrm{AS}}\left(1-\delta_{A}\right)\theta_{i}^{B|A}+p_{i}^{\mathrm{BS}}\left(1-\delta_{B}\right)\left(1-\theta_{i}^{A|B}\right). \end{array}\right.$$

Equation (21) defines the fixed point of the competitive information subsystem in the disease-free state, where all infection probabilities are zero. For threshold calculations, we first solve for a stable information fixed point iteratively under the specified information transmission parameters, weighted network structure, and higher-order interactions. The resulting node-level information probabilities are then used to analyze the linear stability of infection perturbations. If multiple stable information fixed points exist, the calculated threshold is understood as the local invasion threshold associated with the selected stable disease-free information equilibrium.

This procedure does not require a separation of timescales between information and epidemic spreading. Both layers retain their synchronous updates in the full coupled dynamics. The contribution of infection-induced information perturbations to new infections contains products of information and infection perturbations and therefore does not enter the first-order infection spreading matrix. In the following threshold equations, $p_{i}^{\mathrm{US}}$, $p_{i}^{\mathrm{AS}}$, and $p_{i}^{\mathrm{BS}}$ are evaluated at the selected disease-free fixed point, with $p_{i}^{A}=p_{i}^{\mathrm{AS}}$ and $p_{i}^{B}=p_{i}^{\mathrm{BS}}$. This use of a disease-free information fixed point to calculate the epidemic threshold is also consistent with ref. [12].

Using Equation (21) and the definitions of $\tau_{i}^{X}$, the stationary equation for infection perturbations reduces, to first order, to

(22) $$\mu \epsilon_{i}=p_{i}^{\mathrm{US}}\tau_{i}^{U}+p_{i}^{\mathrm{AS}}\tau_{i}^{A}+p_{i}^{\mathrm{BS}}\tau_{i}^{B}.$$

Substituting the full pairwise infection probabilities gives

(23) $$\mu \epsilon_{i}=\sum_{j}\left[p_{i}^{\mathrm{US}}T_{ji}^{U}\left(\beta \right)+p_{i}^{\mathrm{AS}}T_{ji}^{A}\left(\beta \right)+p_{i}^{\mathrm{BS}}T_{ji}^{B}\left(\beta \right)\right]\epsilon_{j}.$$

At the disease-free fixed point, $p_{i}^{\mathrm{US}}=1-p_{i}^{A}-p_{i}^{B}$. Hence, the preceding equation can be rewritten as

(24) $$\begin{array}{cc} \mu \epsilon_{i}=\sum_{j}[ & \left(1-p_{i}^{A}-p_{i}^{B}\right)T_{ji}^{U}\left(\beta \right)\\ \; & +p_{i}^{A}T_{ji}^{A}\left(\beta \right)+p_{i}^{B}T_{ji}^{B}\left(\beta \right)]\epsilon_{j}. \end{array}$$

Since self-loops are absent in the epidemic layer, $b_{ii}=0$. With $d_{ij}$ denoting the entries of the identity matrix, the linearized stationary relation takes the form

(25) $$\sum_{j}\left[K_{ij}\left(\beta \right)-\mu d_{ij}\right]\epsilon_{j}=0,$$

where the effective spreading matrix $K\left(\beta \right)=[K_{ij}\left(\beta \right)]$ is defined by

(26) $$\begin{array}{cc} K_{ij}\left(\beta \right)= & \left(1-p_{i}^{A}-p_{i}^{B}\right)T_{ji}^{U}\left(\beta \right)\\ \; & +p_{i}^{A}T_{ji}^{A}\left(\beta \right)+p_{i}^{B}T_{ji}^{B}\left(\beta \right),\;K_{ii}=0. \end{array}$$

The entry $K_{ij}\left(\beta \right)$ describes the first-order transmission contribution from infected node *j* to susceptible node *i*. The linear update matrix for infection perturbations is (1−μ)I+K(β). Consequently, the local invasion threshold within the MMC framework is determined by

(27) $$\Lambda_{\max}\;\left(K(\beta_{c})\right)=\mu ,$$

where $\Lambda_{\max}$ denotes the largest eigenvalue of the nonnegative matrix $K(\beta_{c})$. Since K(β) retains the full nonlinear dependence of the per-contact infection probabilities on the transmission probability β, the critical transmission probability $\beta_{c}$ is obtained by numerically solving the spectral condition in Equation (27).

Threshold computation comprises solving the disease-free information equilibrium in Equation (21) and numerically finding the root of the spectral condition in Equation (27). Let $L_{\mathrm{info}}$ denote the number of information-equilibrium iterations, $L_{\mathrm{spec}}$ the total number of spectral evaluations, including initialization, and $L_{\mathrm{eig}}$ the average number of power iterations per spectral evaluation. Solving the disease-free information equilibrium requires $O[L_{\mathrm{info}}\left(N+E+S_{2}\right)]$ operations. For each trial value of β, constructing the dense matrix K(β) requires $O(N^{2})$ operations, and each matrix–vector multiplication in the power iteration also requires $O(N^{2})$ operations. The time complexity of threshold computation for a single parameter set is therefore $O\;\left[L_{\mathrm{info}}\left(N+E+S_{2}\right)+N^{2}+L_{\mathrm{spec}}\left(1+L_{\mathrm{eig}}\right)N^{2}\right]$. The separate $O(N^{2})$ term accounts for preprocessing the dense contact background. These iteration counts depend on the model parameters and numerical convergence tolerances. The complexity estimates concern evolution and threshold calculations on a given network and exclude the cost of generating the network beforehand.

Equation (27) shows that the local invasion threshold is jointly determined by the recovery probability, policy-modulated temporal contacts, and the node-level distribution and corresponding protective effects of competing information at the disease-free equilibrium. Weighted interactions and higher-order reinforcement affect population protection through the equilibrium distribution of competing information, whereas temporal contacts and policy intervention affect epidemic spreading through infection opportunities. These effects are jointly reflected in the spectral properties of the effective spreading matrix K(β).

The information-state probabilities $p_{i}^{A}$ and $p_{i}^{B}$ entering K(β) are evaluated at the stable disease-free information equilibrium defined by Equation (21). At this equilibrium, infection probabilities are zero, so the branches leading to infected states in the transition trees are inactive, leaving only information-state transitions among US, AS, and BS. Allowing infected individuals to retain information B changes their information-state assignment after infection but leaves the information-transition rules in the disease-free state unchanged. Therefore, $p_{i}^{A}$ and $p_{i}^{B}$ entering K(β) remain unchanged when the same stable disease-free information equilibrium is considered. If infected individuals’ infectivity, recovery probability, and contact mechanism also remain unchanged, the effective spreading matrix governing first-order infection growth and its spectral condition are unchanged. Infection-induced perturbations to the information distribution affect new infections through their products with infection probabilities and therefore contribute only higher-order terms. Consequently, within the present MMC framework, relaxing this assumption does not change the local invasion threshold associated with the selected stable disease-free information equilibrium.

## 4. Simulation Results

To verify the theoretical analysis and examine the effects of competitive information spreading, temporal contacts, and policy intervention on epidemic dynamics, we perform numerical analyses using the MMC approach together with Monte Carlo (MC) simulations. Unless otherwise specified, the upper information layer contains N=1000 nodes, with a target mean edge degree $k_{1}=10$ and an expected mean 2-simplex participation degree $k_{2}=2$. The ER-type network follows the random simplicial complex construction in ref. [25], with each pair of distinct nodes initially connected independently with probability $p_{1}=\left(k_{1}-2k_{2}\right)/\left(N-1-2k_{2}\right)$. For the BA-type network, the base graph is replaced by a BA graph generated through preferential attachment, with each new node connecting to $m_{\mathrm{BA}}=\left(k_{1}-2k_{2}\right)/2=3$ existing nodes. Subsequently, for both network types, each unordered triplet of distinct nodes is independently designated as a 2-simplex with probability $p_{2}=2k_{2}/\left[\left(N-1\right)\left(N-2\right)\right]$, and its missing edges are added. Different simplices may share nodes or edges, and each triplet is considered only once. Only explicitly designated triplets participate in higher-order information transmission; other triangles are not automatically treated as 2-simplices. After all edges have been generated, edge weights are independently sampled from a normal distribution truncated to [0,1], where $\mu_{a}=0.5$ and $\sigma_{a}=0.2$ are the location and scale parameters before truncation, respectively. Simplex weights are calculated according to Equation (3). The BA-type and ER-type information networks used in the topology comparison have average shortest-path lengths of 3.0763 and 3.3031, and average local clustering coefficients of 0.0806 and 0.0579, respectively. These statistics are evaluated on the unweighted topologies of the final networks. The lower epidemic-spreading layer follows an activity-driven mechanism, with activity variables $y_{i}$ drawn from $F_{R}\left(y\right)\propto y^{-\gamma_{R}}$ over $y_{\min}\leq y_{i}\leq 1$ and intrinsic activity levels $b_{i}=\eta_{R}y_{i}$. The default parameters are $m_{R}=10$, $\eta_{R}=1$, $\gamma_{R}=2.1$, and $y_{\min}=0.1$. Let $\rho^{I}$, $\rho^{A}$, and $\rho^{B}$ denote the steady-state fractions of infected nodes and nodes carrying information A and B, respectively. For the steady-state density calculations, the initial MMC probabilities are $p_{i}^{\mathrm{US}}\left(0\right)=0.98$, $p_{i}^{\mathrm{AS}}\left(0\right)=0$, $p_{i}^{\mathrm{BS}}\left(0\right)=0.01$, and $p_{i}^{\mathrm{AI}}\left(0\right)=0.01$; the MC simulations initialize the four states with the corresponding proportions.

### 4.1. Comparison Between MMC and MC Simulations

**Figure 5:**
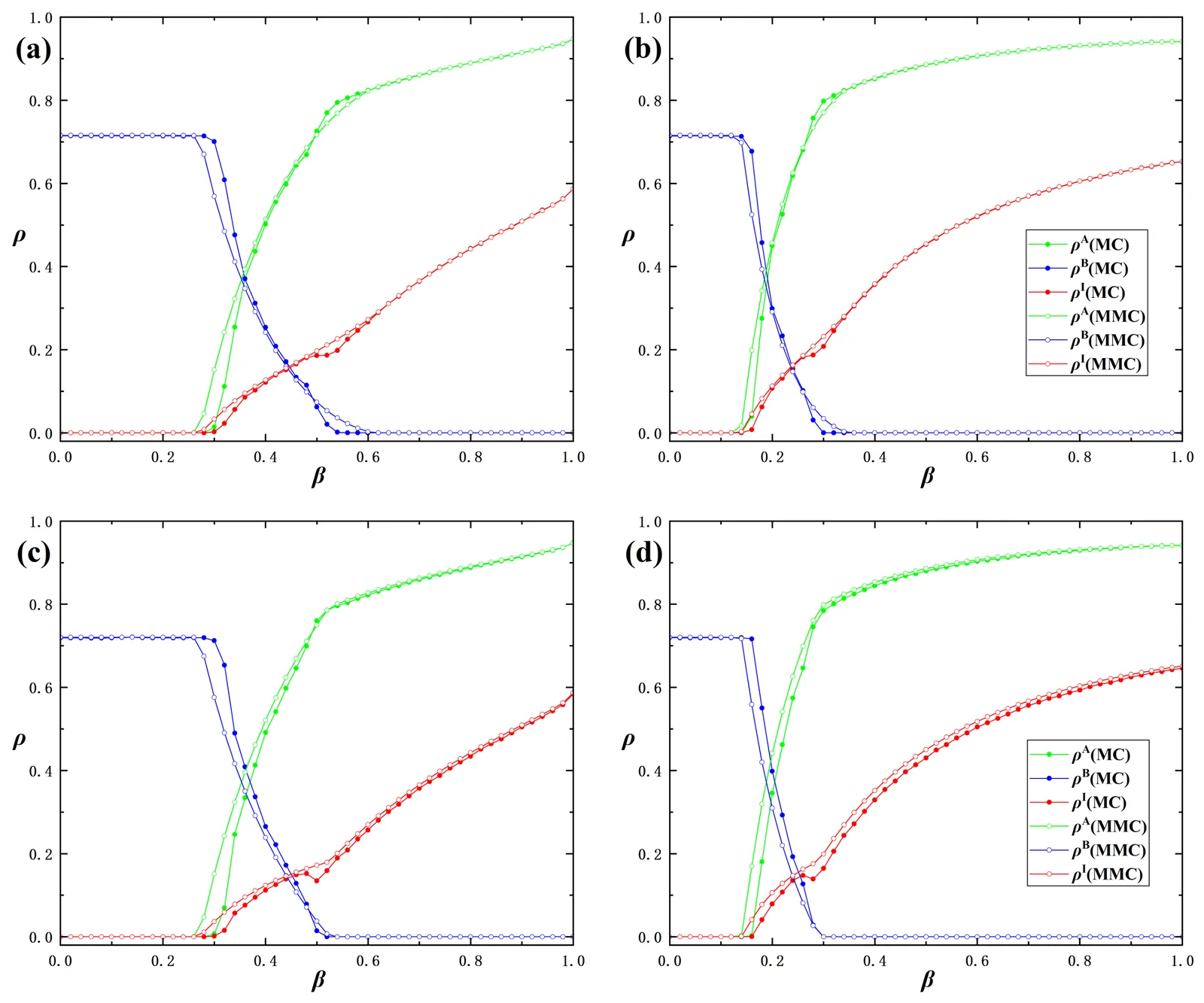
Comparison of the MMC predictions with MC simulation results for the steady-state densities $\rho^{A}$, $\rho^{B}$, and $\rho^{I}$ as functions of the disease transmission rate β. The upper information layer is a BA-type weighted higher-order network in (**a**,**b**) and an ER-type weighted higher-order network in (**c**,**d**). The policy relaxation parameter is set to p=1 in (**a**,**c**) and p=3 in (**b**,**d**). Other parameters are $\lambda_{A}=\lambda_{B}=0.4$, $\lambda_{A}^{*}=\lambda_{B}^{*}=0.5$, $\delta_{A}=\delta_{B}=0.3$, μ=0.3, $\gamma_{A}=0.3$, $\gamma_{B}=0.7$, η=0.3, and α=1. Each MC data point represents the average over 50 simulation realizations for the corresponding parameter setting.

As presented in Figure 5, the theoretical predictions derived from the MMC approach agree well with the MC simulation results under different network topologies and policy parameter settings. For the equilibrium densities associated with information A, information B, and infected nodes, the MMC curves almost overlap with the MC data points over the entire range of β. The agreement remains satisfactory even in the critical regions where the state densities vary rapidly. Such consistency is observed for both BA- and ER-type weighted higher-order networks and for different values of *p*, demonstrating that the developed MMC formulation can effectively reproduce the coupled dynamics among competitive information spreading, temporal contacts, and epidemic transmission. The results also indicate that the theoretical framework is applicable across different network structures and policy conditions.

As the epidemic transmission probability β increases, the system exhibits pronounced coupled information–epidemic evolution. For small β, the epidemic cannot persist and $\rho^{I}$ remains close to zero; in this regime, information B dominates, whereas the density of information A remains low. Once β exceeds the critical value, the prevalence of infection begins to increase continuously and simultaneously promotes the rapid diffusion of the more protective information A, leading to a pronounced increase in $\rho^{A}$. In contrast, $\rho^{B}$ decreases rapidly and eventually approaches zero, reflecting both the feedback of epidemic spreading on protective information diffusion and the competitive replacement between the two types of information. A comparison among the panels further shows that increasing the policy relaxation parameter *p* from 1 to 3 shifts the region of rapid state variation toward smaller β and generally leads to a higher infection density at the same β. Under the present definition of the policy parameter, this result indicates that *p* significantly regulates the epidemic threshold and final prevalence by modifying the effective contact weights in the epidemic layer. These results highlight increased transmission risk during contact restoration and suggest coordinating the relaxation of restrictions with protective information dissemination. By comparison, the overall evolutionary curves for the BA and ER networks remain relatively similar, with only modest differences in the critical region and steady-state densities, suggesting that under the current parameter settings, changes in temporal contacts induced by the policy parameter have a stronger influence than variations in the topology of the upper network layer.

### 4.2. Influence of Competing Information on Disease Prevalence

**Figure 6:**
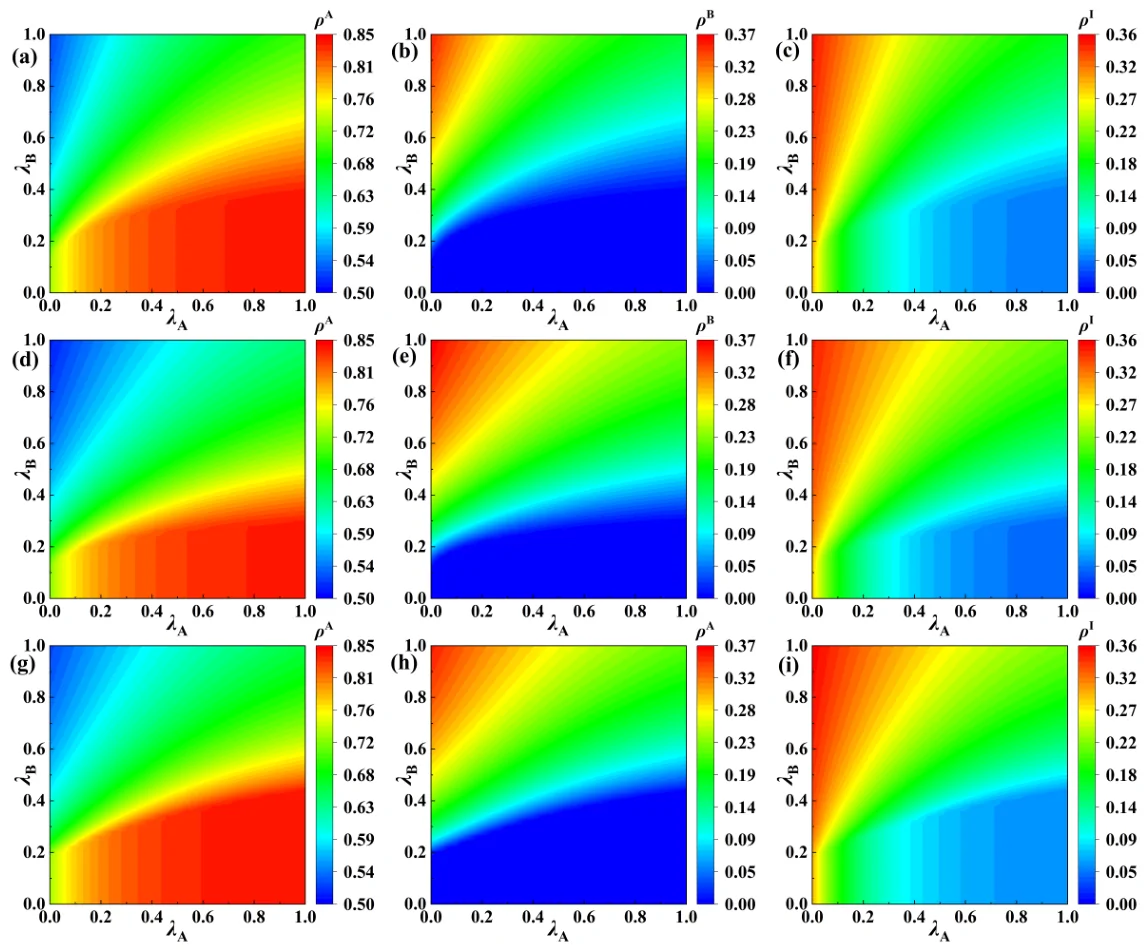
Effects of the two competing information transmission probabilities $\lambda_{A}$ and $\lambda_{B}$ on the equilibrium state densities of the system for varying information preferences and network topologies. (**a**,**d**,**g**) Steady-state density $\rho^{A}$ of information A; (**b**,**e**,**h**) steady-state density $\rho^{B}$ of information B; and (**c**,**f**,**i**) equilibrium density $\rho^{I}$ of infected nodes. The upper information layer adopts a BA-type weighted higher-order network in (**a**–**f**) and an ER-type weighted higher-order network in (**g**–**i**). The information preference parameter is set to α=0.5 in (**a**–**c**) and α=1 in (**d**–**i**). The remaining parameters are $\lambda_{A}^{*}=\lambda_{B}^{*}=0.3$, $\delta_{A}=\delta_{B}=0.2$, μ=0.3, $\gamma_{A}=0.3$, $\gamma_{B}=0.7$, β=0.5, η=0.3, and p=1.

Figure 6 depicts the combined influence of the transmission probabilities $\lambda_{A}$ and $\lambda_{B}$ of two competing types of information on their steady-state distributions and epidemic prevalence, while further considering the roles of the information preference parameter α and upper-layer network topology. Overall, increasing $\lambda_{A}$ substantially increases the steady-state density $\rho^{A}$ of the more protective information A, while both $\rho^{B}$ and the infected density $\rho^{I}$ decrease. In contrast, increasing $\lambda_{B}$ strengthens the competitive advantage of information B, reduces the spreading space available to information A, and maintains the epidemic at a higher prevalence. This opposite regulation arises from the different protective effects of the two types of information, with $\gamma_{A}<\gamma_{B}$ indicating that information A provides stronger protection against infection. Hence, the final epidemic prevalence depends not only on the overall intensity of information spreading but also on which type of information dominates the competitive process. Clear but smooth nonlinear boundaries are observed between different steady-state regimes, without evident abrupt jumps, indicating predominantly continuous transition or crossover behavior within the parameter range considered. In particular, when the two types of information have comparable competitive strengths, relatively small variations in $\lambda_{A}$ or $\lambda_{B}$ can rapidly redistribute their prevalence and consequently induce pronounced changes in epidemic spreading.

A comparison of Figure 6a–c with Figure 6d–f further reveals how the information preference parameter α substantially reshapes the competitive balance between the two types of information. Since α characterizes the preference for information B when an individual is simultaneously exposed to both types, increasing α from 0.5 to 1 enhances the competitive advantage of information B. This weakens the effective coverage of the more protective information A, lowers the overall protection level of the population, and shifts the epidemic toward a higher-prevalence regime. Thus, even when the basic transmission parameters remain unchanged, individual information preference can markedly influence epidemic dynamics by altering the outcome of information competition. In addition, comparing Figure 6d–f with Figure 6g–i reveals broadly similar phase patterns for the BA- and ER-type weighted higher-order networks, with differences mainly appearing as quantitative shifts in the competitive boundaries and steady-state densities. This suggests that network topology modulates the spreading process without changing its fundamental evolutionary pattern, whereas information preference has a more direct effect on the competitive balance. Therefore, effective epidemic mitigation requires not only enhancing the diffusion of highly protective information but also reducing the competitive advantage of less protective information, while network topology further regulates the continuous transition boundaries and final epidemic prevalence through its influence on effective information coverage.

**Figure 7:**
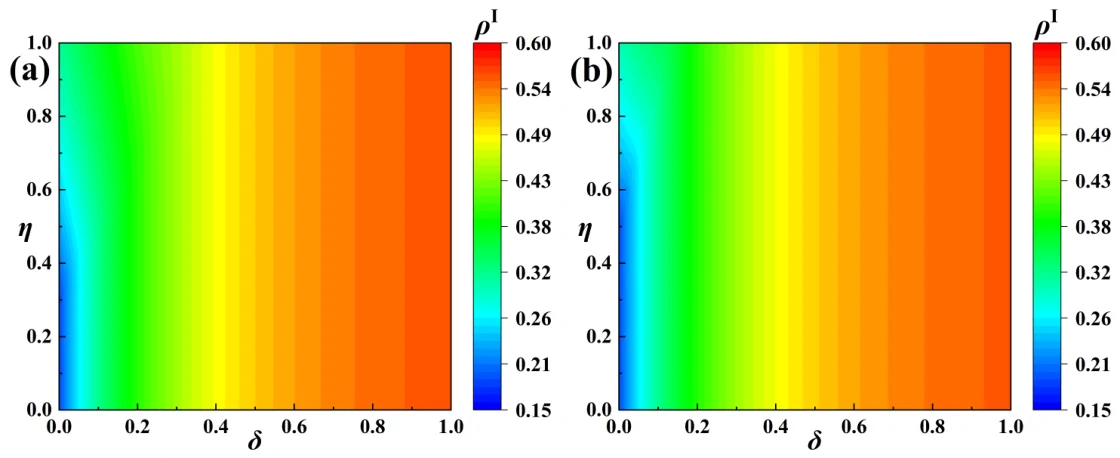
Steady-state infection density $\rho^{I}$ as a function of the common information forgetting probability $\delta =\delta_{A}=\delta_{B}$ and the competition attenuation factor η. The upper information layer is a BA-type weighted higher-order network in (**a**) and an ER-type weighted higher-order network in (**b**). The results are obtained using the MMC method. Both δ and η are varied over [0,1] with a step size of 0.02. The fixed parameters are β=0.5, p=3, $\lambda_{A}=\lambda_{B}=0.4$, $\lambda_{A}^{*}=\lambda_{B}^{*}=0.5$, μ=0.3, $\gamma_{A}=0.3$, $\gamma_{B}=0.7$, and α=1.

To further examine the joint effects of information forgetting and competitive attenuation, Figure 7 shows the steady-state infection density in the δ–η parameter plane. For both information networks, the infection density generally increases with δ, consistent with faster information forgetting weakening the sustained coverage of protective information. In contrast, the effect of η depends strongly on δ: increasing η generally raises the infection density at small δ, whereas the variation along the η direction is weaker at large δ. A larger η reduces the suppression of competing information by an individual’s existing information, thereby facilitating information replacement. Its net effect on infection density therefore depends on the population protection generated by the two information types. The observed pattern is consistent with rapid forgetting shortening information retention and reducing the additional influence of competitive replacement. Despite quantitative differences between the two topologies, the overall trends remain consistent, indicating similar joint effects of information forgetting and competitive attenuation in the BA and ER information networks under the examined conditions. The protective coefficients of the two information types and the epidemic parameters remain fixed throughout this comparison. The dependence of the response to η on δ therefore shows how information retention interacts with competitive replacement to change population protection. This result identifies how information persistence conditions the epidemic effects of competition beyond the relative ordering of the two protective coefficients.

### 4.3. Effects of Higher-Order Interactions on Epidemic Prevalence

**Figure 8:**
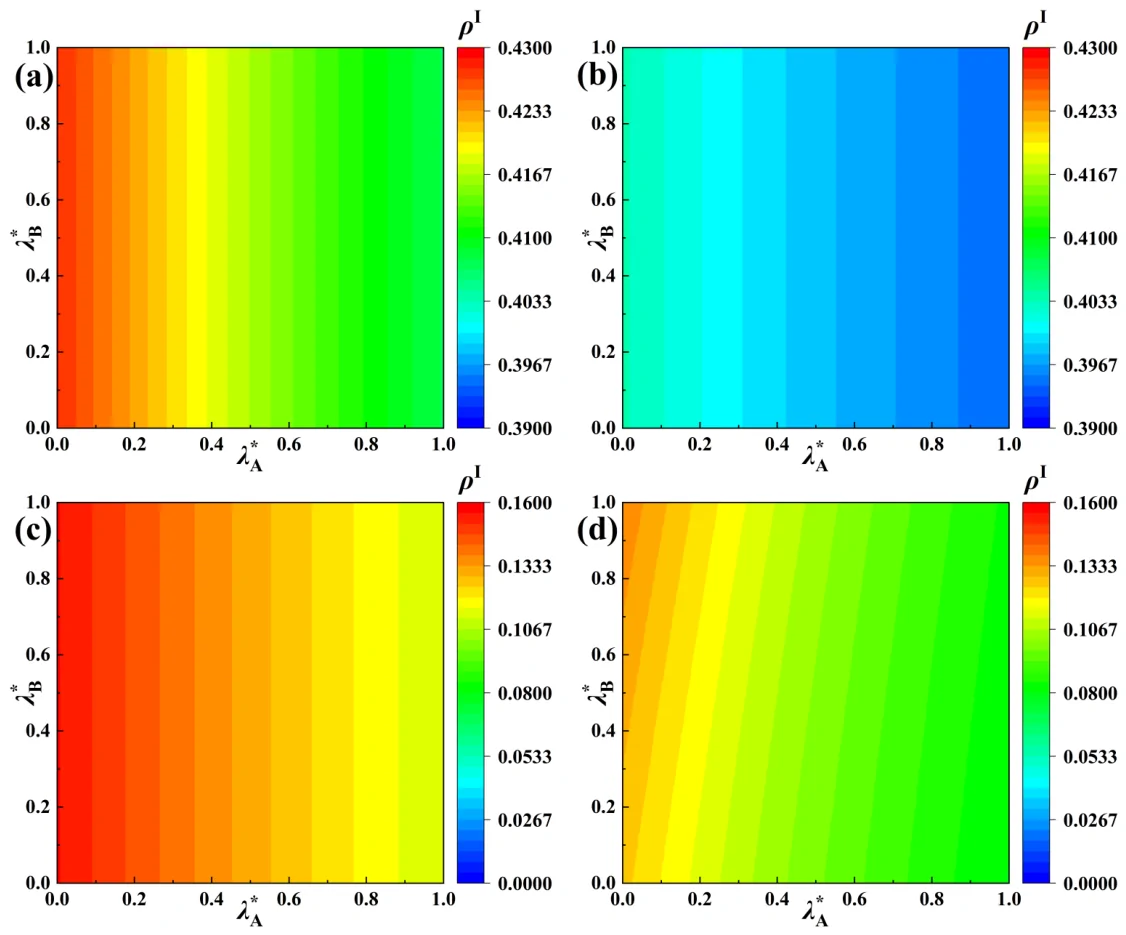
Equilibrium density of infected nodes $\rho^{I}$ as a function of higher-order spreading probabilities $\lambda_{A}^{*}$ and $\lambda_{B}^{*}$ under different mean link weights and activity levels. The upper information-spreading layer is a BA-type weighted higher-order network in all panels. The location parameter of the normal distribution before truncation is set to $\mu_{a}=0.2$ for (**a**,**c**) and $\mu_{a}=0.8$ for (**b**,**d**), while the expected count of links created by an active node is $m_{R}=10$ for (**a**,**b**) and $m_{R}=5$ in (**c**,**d**). Other parameters are $\lambda_{A}=\lambda_{B}=0.3$, $\delta_{A}=\delta_{B}=0.2$, μ=0.3, $\gamma_{A}=0.3$, $\gamma_{B}=0.7$, β=0.5, η=0.3, α=0.5, and p=3.

Figure 8 shows a pronounced asymmetry in the effects of higher-order information spreading on the steady-state infection density: increasing $\lambda_{A}^{*}$ generally reduces infection density, whereas the variation along the $\lambda_{B}^{*}$ direction is relatively weak. This asymmetry arises not only from the different protective effects represented by $\gamma_{A}<\gamma_{B}$, but also from competitive information replacement. For a given configuration of neighbors’ information states, a larger $\lambda_{A}^{*}$ increases the probabilities that unaware individuals adopt information A and that individuals holding B switch to A. Thus, stronger higher-order spreading of A can both expand the coverage of more protective information and reduce the sources of B through competitive replacement. Increased adoption of A may also produce more pairs of A-state neighbors within 2-simplices, further reinforcing its higher-order spreading. These feedback mechanisms can amplify the asymmetric effects of the two information types on epidemic spreading. In contrast, stronger higher-order spreading of B has two opposing effects: adoption by unaware individuals provides some protection, whereas replacement of A by B weakens existing protection. These effects may partially offset each other, helping explain the weaker response to $\lambda_{B}^{*}$ under the examined conditions. The patterns in Figure 8 therefore reflect both differences in protective effects and the competitive redistribution of information, with infection density varying smoothly over the examined parameter range.

Further comparison among the different parameter combinations shows that the mean link weight $\mu_{a}$ and the temporal contact intensity $m_{R}$ exert distinct regulatory effects on epidemic spreading. Contrasting Figure 8a with Figure 8b, together with Figure 8c with Figure 8d, shows that, for the same $m_{R}$, increasing $\mu_{a}$ further reduces the steady-state infection density. This indicates that stronger social interactions facilitate the dissemination and coverage of protective information in the higher-order network, thereby enhancing epidemic suppression. Meanwhile, contrasting the results in Figure 8a,b with those in Figure 8c,d indicates that reducing $m_{R}$ markedly decreases the overall infection level, demonstrating that fewer contacts generated by active nodes within each time step directly weaken the physical transmission pathways of the disease. In comparison, higher-order information spreading mainly reduces infection risk indirectly by strengthening collective diffusion of protective information, whereas $m_{R}$ acts directly on the temporal contact process in the epidemic layer. These results reveal a cross-layer synergistic mechanism for epidemic control: higher-order information spreading in the upper layer enhances population-level protection, while reduced temporal contacts in the lower layer limit effective transmission opportunities, and their combined effects determine the final steady-state epidemic prevalence.

### 4.4. Effects on the Epidemic Threshold

**Figure 9:**
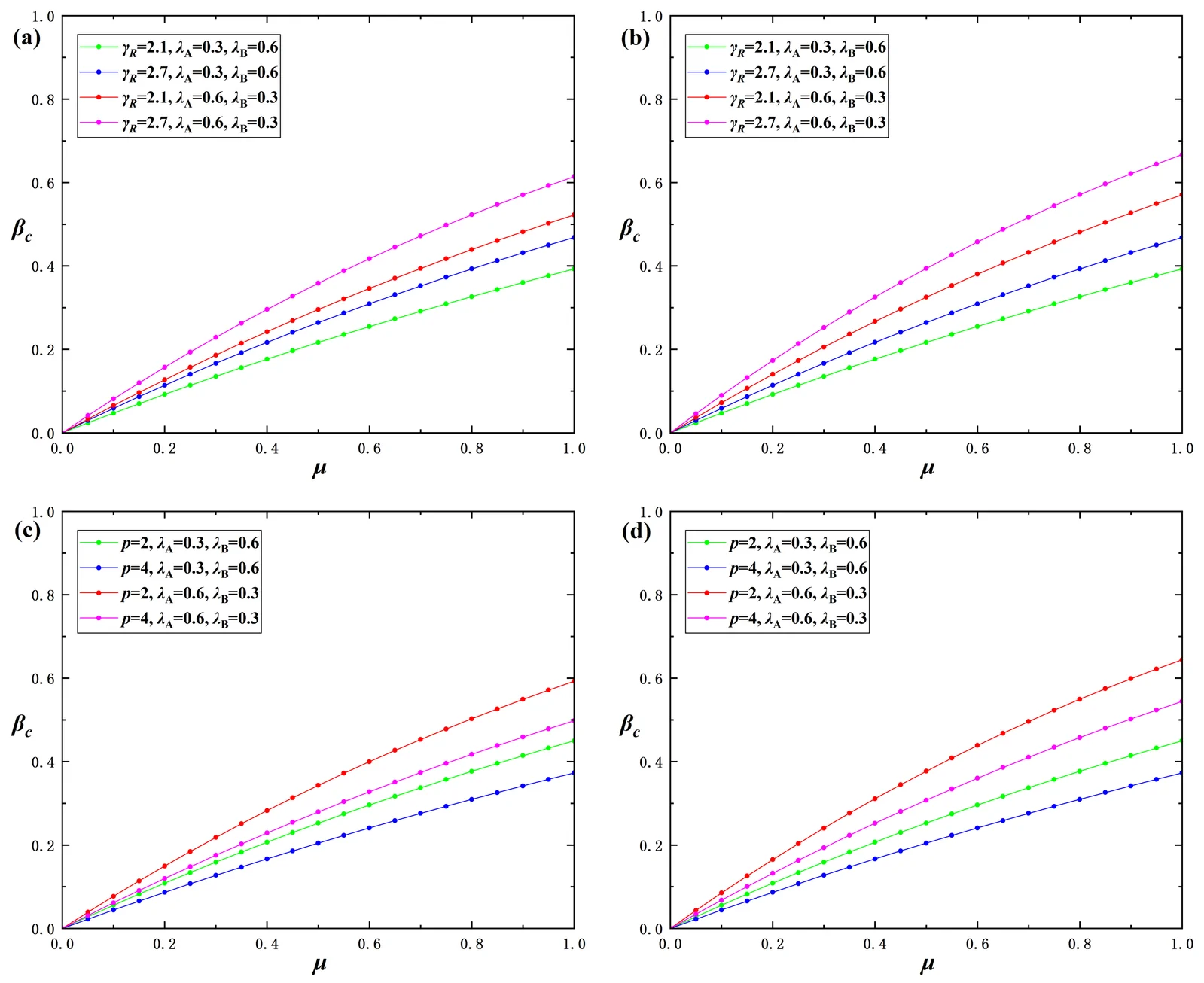
Threshold for epidemic onset $\beta_{c}$ versus the recovery probability μ under different information transmission rates, activity heterogeneity, and degrees of policy relaxation. The upper information-spreading layer is a BA-type weighted higher-order network in (**a**,**c**) and an ER-type weighted higher-order network in (**b**,**d**). In (**a**,**b**), the policy relaxation parameter is fixed at p=3, while the activity exponent is varied as $\gamma_{R}=2.1$ and 2.7. In (**c**,**d**), $\gamma_{R}=2.1$ is fixed, while the policy relaxation parameter is varied as p=2 and 4. In each panel, two combinations of pairwise information spreading probabilities, $\left(\lambda_{A},\lambda_{B}\right)=\left(0.3,0.6\right)$ and (0.6,0.3), are considered. The remaining parameters are $\lambda_{A}^{*}=\lambda_{B}^{*}=0.5$, $\delta_{A}=\delta_{B}=0.3$, η=0.3, α=1, $\gamma_{A}=0.4$, and $\gamma_{B}=0.8$.

Figure 9 shows how the critical transmission probability $\beta_{c}$ varies with the recovery probability μ under different information-spreading, activity, and policy conditions. All curves are obtained by numerically solving the spectral condition in Equation (27), with node-level information probabilities evaluated at the disease-free information equilibrium defined by Equation (21). In all panels, $\beta_{c}$ increases monotonically with μ, while its slope decreases slightly as μ increases, reflecting the nonlinear spectral relation obtained with the full infection probabilities. A higher recovery probability therefore requires a larger transmission probability to sustain infection growth. Under the same activity and policy conditions, $\left(\lambda_{A},\lambda_{B}\right)=\left(0.6,0.3\right)$ consistently yields a higher $\beta_{c}$ than (0.3,0.6). Since information A provides stronger protection, favoring its dissemination relative to information B improves population protection and increases $\beta_{c}$. Figure 9a,b further shows that increasing the activity exponent $\gamma_{R}$ from 2.1 to 2.7 raises $\beta_{c}$. A larger $\gamma_{R}$ concentrates the activity distribution toward lower activity levels, reducing effective contact opportunities.

Comparing the two information networks in Figure 9 shows that the ER information network yields a higher $\beta_{c}$ than the BA information network when $\left(\lambda_{A},\lambda_{B}\right)=\left(0.6,0.3\right)$, whereas their threshold curves are close when $\left(\lambda_{A},\lambda_{B}\right)=\left(0.3,0.6\right)$. This indicates that the influence of topology depends on the protection distribution generated by information competition. The more homogeneous connectivity of the ER network may provide more evenly distributed opportunities to receive information, facilitating the dissemination of protective information across more nodes. In the BA network, stronger degree heterogeneity produces more uneven information-input opportunities. This offers a possible mechanistic explanation for the observed threshold difference when information A has a spreading advantage. According to the definition of the effective infection matrix K(β), replacing U or B by A reduces the corresponding effective infection probabilities under unchanged contact conditions, since $\gamma_{A}<\gamma_{B}<1$. Such changes can lower the dominant eigenvalue at a fixed β and increase the critical transmission probability satisfying Equation (27). Classical studies of static networks establish the importance of connectivity heterogeneity for epidemic thresholds [11], while information–epidemic coupling studies show that node-level protective information distributions can modify these thresholds [12]. In the present model, ER/BA topology varies in the upper information layer, and its influence is transmitted to the lower activity-driven spreading process through the disease-free information distribution. The threshold difference should therefore be interpreted through the full node-level information probabilities and contact structure, within the examined competitive-information conditions.

Figure 9c,d shows the influence of the policy relaxation parameter *p* on $\beta_{c}$. For both combinations of information transmission probabilities, increasing *p* from 2 to 4 reduces $\beta_{c}$. Under the policy mechanism defined in this model, a larger *p* corresponds to less restrictive contact conditions and increases effective contact intensity, allowing epidemic invasion at a lower transmission probability. This trend is consistent across the BA and ER information networks. Meanwhile, at the same *p*, $\left(\lambda_{A},\lambda_{B}\right)=\left(0.6,0.3\right)$ still yields a higher $\beta_{c}$, indicating that favoring the more protective information helps inhibit epidemic invasion under different policy conditions. These results show that recovery probability, individual activity distributions, policy regulation, and competitive information jointly affect $\beta_{c}$. Information spreading and information-layer topology modify population protection through the disease-free information distribution, while activity-driven contacts and policy regulation directly modify infection opportunities. Together, these pathways determine the local invasion condition.

## 5. Conclusions

In this study, we developed a bilayer competitive information–epidemic coupled propagation framework that integrates weighted higher-order information interactions, activity-driven temporal contacts, and policy regulation within a unified framework. The upper layer includes pairwise spreading together with 2-simplex-driven higher-order group spreading, with two competing types of information providing different levels of protection, while the lower layer is represented by a weighted activity-driven network to capture dynamically changing physical contacts. Based on the MMC approach, we formulated node-level evolution equations and derived a spectral condition for the local invasion threshold $\beta_{c}$, which was evaluated numerically. The MC simulations showed good agreement with the MMC predictions for the steady-state densities. The numerical findings indicate that enhancing the diffusion of the more protective information A increases its steady-state coverage, reduces epidemic prevalence, and raises the epidemic threshold, whereas a stronger preference for the less protective information B weakens the overall protective effect and facilitates epidemic spreading. Higher-order interactions produce a clearly asymmetric impact on the two competing information types, with stronger higher-order spreading of information A producing a more pronounced suppressive effect on the epidemic. In addition, stronger social ties facilitate the diffusion of protective information, whereas reducing the number of contacts generated by active individuals directly weakens epidemic transmission pathways. The epidemic threshold is further jointly regulated by the recovery probability, activity heterogeneity, information-layer topology, and policy-modulated contact intensity; under the policy mechanism defined in this study, a larger *p* represents greater policy relaxation and increases the effective contact intensity and consequently lowers the epidemic threshold. These model results suggest coordinating the relaxation of contact restrictions with the dissemination of highly protective information and the management of effective contact intensity. These results are consistent with earlier awareness–epidemic studies showing that protective awareness can influence epidemic invasion [12], and further clarify how this effect depends on the node-level protection distribution generated by two competing information types. Combining simplicial group reinforcement [25] with activity-driven temporal contacts [30], the present study identifies two interconnected pathways: information competition changes infection risk through the protection distribution, while individual activity and policy regulation change disease exposure through effective contacts.

The model assumes that infected individuals immediately recognize their infection and adopt information A, which may overestimate protective information feedback in populations with delayed responses or continued adoption of information B during infection. In addition, the SIS framework omits latent stages and post-recovery immunity, while the restriction to two mutually exclusive information types with fixed protective effects does not capture multiple coexisting information sources or differences in individual trust. Although node activities and contact weights are heterogeneous, policy regulation is controlled by a common macroscopic parameter *p*, without distinguishing policy measures and responses across regions or population groups. The present evaluation mainly relies on comparisons between MMC predictions and MC simulations, without parameter calibration or empirical validation using real social or epidemiological data. Empirical validation of the full coupled framework requires linking epidemic observations to competing information adoption, group interactions, contact behavior, and policy changes; case time series alone would provide limited constraints on these mechanisms. Future research could incorporate delayed and heterogeneous information responses, allow more general information states during infection, and consider how these states affect contact behavior and infectivity. Other extensions include SEIR or SEIRS disease frameworks, multiple coexisting information types, group-specific or adaptive policy interventions, and network adaptation in response to information and disease states. For empirical research, real epidemic data could be combined with corresponding information diffusion data, contact observations, and policy records for parameter calibration. Model predictions could then be evaluated against independent observations to assess the explanatory and predictive capabilities of the framework for specific epidemics.

## Data Availability

All data that support the findings of this study are included within the article.
